# Editorial: A Multidisciplinary Look at *Stenotrophomonas maltophilia*: An Emerging Multi-Drug-Resistant Global Opportunistic Pathogen

**DOI:** 10.3389/fmicb.2017.01511

**Published:** 2017-08-31

**Authors:** Joanna S. Brooke, Giovanni Di Bonaventura, Gabriele Berg, José-Luis Martinez

**Affiliations:** ^1^Department of Biology, DePaul University Chicago, IL, United States; ^2^Department of Medical, Oral, and Biotechnological Sciences, Center of Excellence on Aging and Translational Medicine (CeSI-MeT), “G. d'Annunzio” University of Chieti-Pescara Chieti, Italy; ^3^Institute of Environmental Biotechnology, Graz University of Technology Graz, Austria; ^4^Biotecnologia Microbiana, Centro Nacional de Biotecnología (CSIC) Madrid, Spain

**Keywords:** *Stenotrophomonas maltophilia*, antibiotic resistance mechanisms, cystic fibrosis, biofilms, iron, antimicrobial activity, biocontrol, bioremediation

*Stenotrophomonas maltophilia* is a human opportunist pathogen, with an environmental origin. Members of the species are common inhabitants of water and soils, including rhizosphere. *S. maltophilia* can be a plant endosymbiont and is found in animals and washed foods. This Gram-negative bacterium has intrinsic resistance to various classes of antimicrobials. Within the *S. maltophilia* genome, genes encoding antibiotic inactivating enzymes, multidrug efflux pumps, and a quinolone resistance gene contribute to its reduced antibiotic susceptibility. Although a low virulence pathogen, *S. maltophilia* can cause various infections in susceptible patients. In addition, *S. maltophilia* isolates present important biotechnological properties, which can be carefully taken into consideration given the pathogenic potential of this microorganism. This research topic examines *S. maltophilia* from different perspectives, and it includes 11 articles: 1 commentary, 7 primary research articles, and 3 review articles.

In the first article, Berg and Martinez provide a global perspective on the two faces of *S. maltophilia*: a bacterial pathogen as well as an organism that can be used as a biocontrol agent or stress protective agent in crop production, and in bioremediation. They highlight the difficulties distinguishing between beneficial and harmful strains of *S. maltophilia*, and suggest *Stenotrophomonas rhizophila* as a close relative and harmless alternative for use in biotechnology. *S. rhizophila*, is a plant-associated organism that grows only at a lower temperature than *S. maltophilia*, suggesting its inability to cause disease in humans.

## Genomes and biology

The genetic and metabolic diversity of *S. maltophilia* reflects its diverse habitats. In their review, Mukherjee and Roy describe the intra- and inter-species genetic diversity of *S. maltophilia* strains. The metabolic diversity of *S. maltophilia* is responsible for production of novel bioactive compounds including biocontrol agents against microbes and insects, enzymes and nanoparticles used in medicinal, industrial, and bioremediation applications. This article reviews the use of *S. rhizophila* in phyto- and rhizo-remediation.

Pompilio et al. examine the phenotypic and genotypic diversity of *S. maltophilia* during a decade-long colonization in the lungs of a cystic fibrosis (CF) patient. Two distinct groups are present among the 13 temporally isolated *S. maltophilia* strains. The study demonstrates that *S. maltophilia* adapts to the CF lung with increased growth rate and antibiotic resistance, but with lowered biofilm formation and decreased pathogenicity. During chronic CF lung infection, *S. maltophilia* develops new phenotypes, likely due to genetic or epigenetic changes.

## Antibiotic resistance and pathogenesis

*S. maltophilia* has intrinsic resistance to various antibiotics. Sanchez in her review provides updated information about the molecular mechanisms underlying resistance: efflux pumps, low membrane permeability, antibiotic-modifying enzymes, or the quinolone resistance gene. *S. maltophilia* can acquire drug resistance via mutations and horizontal gene transfer. The understanding of the intrinsic resistome of *S. maltophilia* is currently limited. Sanchez reports on phenotypic resistance (without genotypic changes) that can occur in *S. maltophilia* including biofilm production. This review highlights the need for new drugs to thwart *S. maltophilia* infections.

In their review, Chang et al. provide information about prevalence rates of infection due to *S. maltophilia*, coming from nationwide and worldwide surveillance. They report on the prevalence of *S. maltophilia* in intensive care units, pediatric populations and in community-acquired infections. The antimicrobial susceptibility of *S. maltophilia* is presented, as well as molecular resistance mechanisms and current drug treatment strategies.

To investigate how quinolone resistance in *S. maltophilia* can occur through molecular mechanisms other than mutation of genes encoding bacterial topoisomerases, Bernardini et al. used a transposon mutagenesis approach. They observe that when a gene encoding for RNase G is inactivated, this *S. maltophilia* mutant displays reduced susceptibility to quinolones. Complementation of the mutant with the wild type RNase G gene restores quinolone sensitivity. Inactivation of RNase G results in increased expression of genes involved in *S. maltophilia* heat shock response. The study shows that heat shock induces a transient phenotype of quinolone resistance in *S. maltophilia*. This study further demonstrates that additional molecular mechanisms are used to acquire antimicrobial resistance.

Roscetto et al. found that *S. maltophilia* strains (from CF and non CF patients and from the environment) demonstrate heterogeneity in their ability to internalize and replicate within human monocyte-derived dendritic cells (DCs). All strains activated DCs, as measured increases in surface maturation markers and proinflammatory cytokines (TNFα and IL-12) were observed. No significant differences in the maturation of immature DCs were observed between the strains.

The virulence of *S. maltophilia* is controlled by iron availability, probably using the Fur system, as described by Garcia et al. This study reports that biofilm formation, oxidative stress response, outer membrane proteins expression, and diffusible signal factor (DSF) production are mediated by iron availability. Confocal laser scanning microscopy shows that iron depletion stimulated biofilm formation resulting in low reactive oxygen species production. This study shows that iron also negatively regulates DSF production in *S. maltophilia* and that a spontaneous *fur* mutant is more virulent in a *Galleria mellonella* infection model.

Huedo et al. have investigated the genetic and functional diversity of the DSF quorum-sensing machinery in *S. maltophilia*. The *rpf* cluster controls this system in *S. maltophilia*. Two variants, *rpf*-1 and *rpf*-2, are used to distinguish between two *S. maltophilia* groups which may have originated through horizontal exchange. The *rpf*-1 strains make DSF whereas the *rpf*-2 strains produce DSF only in the presence of DSF-producers. The production of DSF is mediated by temperature, culture media composition, and fatty acid supplementation. This article suggests that DSF is produced through cross-talk between *rpf*-1 and *rpf*-2 strains and using a positive-feedback mechanism. The *rpf*-1 and *rpf*-2 strains act synergistically to promote virulence in the zebrafish infection model.

It has been suggested that in co-culture biofilms, *S. maltophilia* modulates the virulence of *Pseudomonas aeruginosa* (Pompilio et al.). To further understand the interactions of these bacteria, each pathogen was co-isolated from one chronically infected CF patient. Each strain was assessed during planktonic growth, adhesion and biofilm formation, motility, and gene expression in mixed biofilms. *P. aeruginosa* inhibits *S. maltophilia*'s growth planktonically and in biofilm. *S. maltophilia* affects *P. aeruginosa*'s ability to adhere to CF bronchial cells and induces alginate over-production by *P. aeruginosa*. The alginate over-production may have been responsible for the decreased susceptibility of *S. maltophilia* to tobramycin in mixed culture biofilms. Understanding these interspecies interactions can lead to development of novel therapeutic strategies against these pathogens.

## *S. maltophilia* and biotechnology

Mukherjee and Roy present *S. maltophilia* as a useful biocontrol agent against fungi, bacteria, and insects. In their review, they note its ability to produce various enzymes for biotechnological applications, including the degradation of keratin, atrazine, trichloroethylene, dichlorodiphenyltrichloroethane (DDT), and its use in metal bioremediation, and its colonization of plant roots. A safer non-pathogenic alternative to using *S. maltophilia* is suggested, *S. rhizophila* (Berg and Martinez).

*S. maltophilia* demonstrates antimicrobial activity. Dong et al. found that *S. maltophilia* produces a phage-tail like bacteriocin, maltocin P28 with broad activity against Gram-positive and Gram-negative bacteria. The activity of purified maltocin P28 is stable across different temperatures and pH, and it causes lysis of the target cell without EDTA treatment. Maltocin P28 demonstrates lytic activity against several pathogenic bacteria.

## Author contributions

JB wrote the manuscript which was then reviewed by GDB, GB, and JM.

### Conflict of interest statement

The authors declare that the research was conducted in the absence of any commercial or financial relationships that could be construed as a potential conflict of interest.

